# Metagenomic interrogation of urban Superfund site reveals antimicrobial resistance reservoir and bioremediation potential

**DOI:** 10.1093/jambio/lxaf076

**Published:** 2025-04-16

**Authors:** Sergios-Orestis Kolokotronis, Chandrima Bhattacharya, Rupobrata Panja, Ian Quate, Matthew Seibert, Ellen Jorgensen, Christopher E Mason, Elizabeth M Hénaff

**Affiliations:** Departments of Epidemiology and Biostatistics, Medicine, and Cell Biology, SUNY Downstate Health Sciences University, 450 Clarkson Ave, Brooklyn, NY, United States; Department of Physiology and Biophysics, Weill Cornell Medicine, 1300 York Avenue 0021, United States; Center for Computational and Integrative Biology, Rutgers University, 201 S Broadway Camden, NJ 08103, United States; Fruit Studio, 352 Depot Street, Suite 250, Asheville, NC 28801, United States; School of Architecture, University of Virginia, Campbell Hall, PO Box 400122, Charlottesville, VA 22904, United States; Biotech without Borders, 43-01 21st St Suite 319, Long Island City, NY 11101, United States; Department of Physiology and Biophysics, Weill Cornell Medicine, 1300 York Avenue 0021, United States; NYU Tandon School of Engineering, Integrated Design and Media, Center for Urban Science and Progress, Chemical and Biomolecular Engineering, 370 Jay Street, Brooklyn, NY 11201, United States

**Keywords:** EPA Superfund, environmental contamination, metagenomics, bioremediation, antimicrobial resistance, biomining

## Abstract

**Aims:**

We investigate the bioremediation potential of the microbiome of the Gowanus Canal, a contaminated waterway in Brooklyn, NY, USA, designated a Superfund site by the US Environmental Protection Agency due to high concentrations of contaminants, including polychlorinated biphenyls, petrochemicals, and heavy metals.

**Methods and results:**

We present a metagenomic analysis of the Gowanus Canal sediment, consisting of a longitudinal study of surface sediment and a depth-based study of sediment core samples. We demonstrate that the resident microbiome includes 455 species, including extremophiles across a range of saltwater and freshwater species, which collectively encode 64 metabolic pathways related to organic contaminant degradation and 1171 genes related to heavy metal utilization and detoxification. Furthermore, our genetic screening reveals an environmental reservoir of antimicrobial resistance markers falling within 8 different classes of resistance, as well as *de-novo* characterization of 2319 biosynthetic gene clusters and diverse groups of secondary metabolites with biomining potential.

**Conclusion:**

The microbiome of the Gowanus Canal is a biotechnological resource of novel metabolic functions that could aid in efforts for bioremediation, AMR reservoir mapping, and heavy metal mitigation.

Impact Statement The Gowanus Canal Superfund site is emblematic of the many post-industrial sites facing a legacy of hazardous pollutants, and its resident microbiome harbors the potential to remediate anthropogenic contaminants.

## Introduction

### Historical background of the gowanus canal

The Gowanus Canal in Brooklyn, NY, USA is an Environmental Protection Agency (EPA) Superfund site (Miller 2015) which contains significant amounts of chemicals of concern (Mandigo et al. 2016). These include heavy metals like arsenic, copper, lead, and iron; halogenated compounds, including polychlorinated biphenyl (PCB); chlorinated solvents and fungicides, including carbon tetrachloride and ethylene dibromide; polycyclic aromatic hydrocarbons (PAH); volatile organic compounds (VOC) and semivolatile organic compounds; petroleum products; and dissolved salts, including brine and other acids such as tartaric acid (U.S. EPA 2011). Following the 2010 designation of the canal as a Superfund site on the National Priorities List, plans were announced to dredge and sub-aquatically cap the waterway over a decade (U.S. EPA 2012) in a multiphase project expected to finish in mid-2025 (Krisel 2015).

### Microbial response to anthropogenic contamination

The Gowanus Canal is an extreme environment with both built and natural components in the most populated city in the U.S. (U.S. Census Bureau 2025), and provides a testbed for studying the impact of engineered environments on resident ecosystems. Studies have shown that microorganisms, especially extremophiles, respond to the challenge of anthropogenic contaminants through adaptive degradation and biosorption (Xu et al. 2010, Ghosh and Das 2018, McCutcheon et al. 2021, Sharma et al. 2021). One such example includes the rapid shifts in marine microbial population observed after the DeepWater Horizon Spill both in open ocean (Mason et al. 2012, Redmond and Valentine 2012, Kimes et al. 2014) and coastal wetlands (King et al. 2015), where the relative abundance of petrochemical-degrading species increased significantly after the spill. In addition to natural adaptation of endogenous species, microbial remediation can occur by introducing target species to an environment. For example, introduction of a consortium consisting of probiotic bacteria efficient in degrading naturally occurring petroleum hydrocarbons to coral environments was shown to be effective in remediating oil spills without negatively impacting the coral holobiont, unlike using chemical dispersants (Fragoso ados Santos et al. 2015). A two-stage fungal remediation study to tackle textile industry dye effluent utilizing both adsorption and accumulation illustrated the potential of integrated approaches in bioremediation (Mathur et al. 2018). Microbial bioremediation, while presenting constraints of efficacy and scale, can be considered preferable over physico-chemical remediation as it does not leave further residues (Das and Dash 2014).

Superfund sites, with their high concentrations of toxic compounds, can be considered an extreme environment and extremophiles are known for their potential for bioremediation (Kaushik et al. 2021). While every Superfund site is unique in its composition of contaminants, a growing body of literature has shown that microbial bioremediation often develops spontaneously. While microbial diversity can be decreased at a toxic site, the abundance of bioremediation-related species increases (Lowe et al. 2002) with specific functions such as the microbial dechlorination of compounds, including PCBs (Rodenburg et al. 2015). Microbial bioremediation is a long standing practice (Antizar-Ladislao 2010), and more recently sequencing technologies and metagenomic analyses have enabled the identification of genes and species responsible for observed remediation-related metabolisms (Dangi et al. 2019, Gupta et al. 2020). These observations, in addition to the accelerated evolution of microbial communities in extreme environments (Li et al. 2014), make Superfund sites candidates for metabolism mining—both with respect to contaminant-related functions, such as metal rare earth element binding, and also for discovery of novel functions or metabolites of potential biotechnological interest. Indeed, microorganisms in the environment can produce a wealth of secondary metabolites (SMs) which have been historically used for various biotechnological applications, including production of antibiotics, anticancer agents, and agrochemicals (Katz and Baltz 2016). SMs synthesized through the expression of biosynthetic gene clusters (BGCs) can be hard to identify using bioactivity screens as BGCs are often silent, only activated under pleiotropic environmental triggers such as growth condition variation or introduction of competing species (Rutledge and Challis 2015). Genome mining using high-throughput sequencing approaches has been used to identify BGCs responsible for encoding these SMs without the need for identifying the appropriate activation triggers (Udwary et al. 2021). In particular, environmental microbiomes, even urban ones, can be a rich substrate for the discovery of such molecules (Charlop-Powers et al. 2016). Given the Gowanus Canal’s unique—and extreme—environment, we hypothesize that its microbiome might be a source of novel BGCs.

To provide a detailed molecular portrait of the microbial adaptations in the Canal, we used shotgun metagenomic sequencing and a suite of bioinformatic tools with the goals to (i) characterize species diversity, (ii) assess potential for bioremediation, and (iii) discover novel BGCs. As a community that has thrived in a highly contaminated environment for over a century and a half, this microbiome may hold keys for living and thriving under uniquely toxic selection pressures as particular to the putative Anthropocene era (Waters and Turner 2022).

## Materials and methods

### Site selection and sample collection

**Figure 1.**
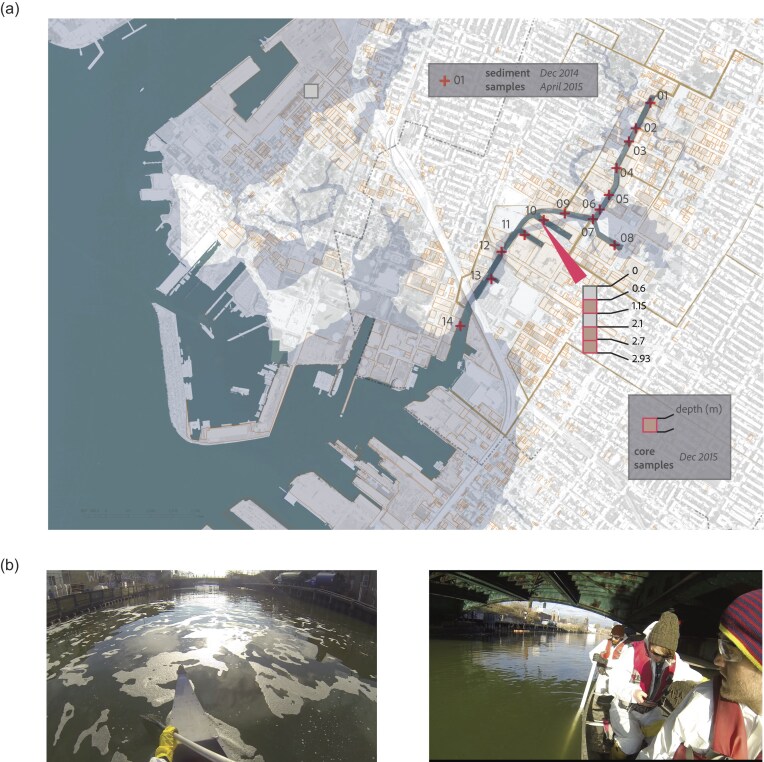
(a) Map of sampling locations along the Gowanus Canal. The 14 surface sediment samples are marked with “+” signs, and the core sample location is marked with an arrow. (b) Process documentation shots of surface sediment sampling. Photo credit: Dave Rife.

Sediment sampling was performed across fourteen sites along the length (2.9 km) of the Canal (numbered 01–14 in Fig. 1). Sediment was sampled from a canoe, using a 4.5 m long PVC (PolyVinyl Chloride) pipe as a sampling device. The pipe was driven approximately 10–15 cm into the sediment, capped, and pulled up to the surface. Upon uncapping, the retained sediment was dispensed into a 50 ml sterile tube. Core samples were obtained through the EPA and private stakeholders during the feasibility study for the EPA Superfund site cleanup. The GPS coordinates of the fourteen sediment sample locations, as well as the core sample location and additional metadata are reported in Table S1.

### Sampling scheme

Samples were collected from the sediment surface as well as one sediment core. Surface sediment samples were collected in 14 different locations along the length of the Canal, at two different timepoints (December 2014 and April 2015). These are samples from the top 15 cm of sediment, under 1–5 m of water. These will be referred to as “sediment samples” throughout this paper. Core samples were collected at a single location, from a core taken 3.5 m deep from the surface of the sediment, in 0.5 m depth increments (Fig. 1a for a map of these locations, and Fig. 1b for images of the process). These were collected from a core taken by the Department of Environmental Protection, and depth refers to the depth under the surface of the sediment. These will be referred to as “core samples.” See sample metadata in Table S1.

### DNA extraction and sequencing

Sediment and core samples were stored at −80°C until processing. DNA was extracted using the MoBio PowerSoil DNA Isolation Kit (catalog no. 12888–50) according to the manufacturer’s instructions, with 250 mg of starting material and lysis step performed on the Qiagen PowerLyzer (3 × 30 s agitation) then purified with Ampure XP beads (Beckman Coulter A63881). Sediment sample libraries were prepared using the Qiagen GeneRead DNA Library I Core Kit (180 432) with the Qiagen GeneRead DNA Library I Amp Kit (180455) along with Qiagen Adapter I Set A 12-plex (180985) and Qiagen Adapter I Set B 12-plex (180986) and sequenced on the Illumina HiSeq 4000 platform. Core sample libraries were prepared with the Roche KAPA HyperPrep Kit (KK8504, 07962363 001) and sequenced on the Illumina NovaSeq 6000 platform. For both platforms, paired-end 150 bp reads were generated, with an average of 26.5 million read pairs (53 million total reads) per sample. Sample metadata, including sequencing platform and read numbers are reported in Table S1.

### Bioinformatics analysis

All bioinformatic analyses were performed on the High Performance Computing cluster at New York University. Preprocessing and AMR was calculated using the MetaSUB CAP pipeline (https://github.com/MetaSUB/MetaSUB_CAP) (Danko et al. 2021). Unless specified otherwise, default settings were used for the tools listed below.

### Preprocessing

Shotgun metagenome raw sequence data, available as pair-end reads, was first processed with AdapterRemoval (v2.2.2) (Schubert et al. 2016), for the removal of low-quality reads and reads with ambiguous bases. This was followed by using Bowtie2 (v2.2.3) (Langmead and Salzberg 2012) for the alignment to the human genome (hg38, including alternate contigs). Read pairs where one or both ends mapped to the human genome were discarded while read pairs where neither mate mapped (referred to as “non-human reads” hereafter) were used for further analysis.

### Statistical analysis and visualization

All statistical analysis and visualization was done in RStudio v4.1.2 (RStudio Team 2020). The statistical tests performed are reported in scripts (see github repository below) as well as figures. Packages used for plotting include Vennerable (Jia et al. 2021), igraph (Csárdi and Nepusz 2006), ggraph (Pedersen 2025), circlize (Gu et al. 2014), ggPlot2 (Wickham 2011), and RainCloudPlots (Allen et al. 2019). The code for the plots is available at https://github.com/HenaffLab/2023_Gowanus_Publication.

### Taxonomic assignment

Non-human reads were used for taxonomic profiling using MetaPhlAn3 (v3.0.7) (Beghini et al. 2021), which produces species-level resolution using clade-specific marker genes for profiling taxonomy. MetaPhlAn was run with the flags “—add_viruses” to include viral markers and “—tax_lev ‘s’” to specify taxonomic resolution at the species level, with taxonomy distributions reported as relative abundance (Table S2).

### Dimensionality reduction

Dimensionality reduction of taxonomic data was performed using the R package Uniform Manifold Approximation and Projection for Dimension Reduction (UMAP) (Becht et al. 2019) as it not only preserves the global structure, but also performs a non-parametric mapping. In order to investigate sequencing depth as a factor in UMAP groupings (core samples were sequenced at higher depth that the Sediment samples, see Table S1 for read depths), we downsampled the core reads to the average sediment read depth to generate a normalized dataset. This normalized dataset also generates a similar UMAP plot as seen in Figure S2, and the full dataset was used for all subsequent analyses.

### Diversity metrics

The R Vegan package (Dixon 2003) was used for calculation of diversity metrics. Alpha diversity was calculated using the Simpson and Shannon (also referred to as Shannon–Wiener) indices. The Simpson index or the dominance index accounts for the proportionality of a species in the sample. The Shannon–Wiener index takes into account the randomness present at the site. It also accounts for both the species’ equitability and richness. We report species richness which refers to the total number of different species present in an ecological community. We use Bray–Curtis dissimilarity to report Beta diversity, which quantifies the compositional differences between two sites, accounting for species counts. We use non-metric multidimensional scaling (NMDS) for visualization of beta diversity using Bray–Curtis dissimilarity.

### Microbial species annotation

We used The Microbe Directory v2 (TMD) (Shaaban et al. 2018, Sierra et al. 2019) to annotate species based on ecological and phenotypical features. TMD is a machine readable and hand curated database consisting of taxonomic annotation of viruses, bacteria, fungi, and eukaryotes. The features we reported include extremophilic nature, type of metabolism, and known microbiome isolation location. Automatic characterization of microbes remains challenging, and the breadth and depth of current TMD results can vary based on emerging literature and as more microbes are characterized and accessioned. We have used TMD v2 for characterizing most of the microbes referred to in Table S3, but for some species, we have collected additional literature review, which is referenced in the body of this manuscript.

### Bioremediation and metabolic studies

Non-human reads were used to characterize metabolic function using the Human Microbiome Project Unified Metabolic Analysis Network3 (HUMAnN3) (v3.0.0.alpha.4) (Beghini et al. 2021), specifying DIAMOND (Buchfink et al. 2015) as the alignment algorithm and UniRef50 (Suzek et al. 2015) as the target database. This tool references the METACYC database for assembling pathways based on genes identified. We utilized the pathway abundances (Table S9) and the pathway coverage files for further analysis (all output files available with supplementary data at https://github.com/HenaffLab/2023_Gowanus_Publication). Pathway abundance values were normalized to copies per million and stratified using the script “humann_infer_taxonomy” provided by HUMAnN3, specifying resolution at the species level (Table S7). Following this, the pathways identified by HUMAnN3 were cross-referenced with the Biocatalysis/Biodegradation Database (http://eawag-bbd.ethz.ch) (Gao et al. 2010), to select pathways with bioremediation potential (Table S8).

### Antimicrobial resistance

We generated AMR profiles by using non-human reads as input to MEGARES2 v1.0.1 using Bowtie2 v2.2.3 (—end-to-end mode, very-sensitive option). MEGARes includes an ontology to group resistance genes according to gene groups (Table S4) and AMR mechanisms (Table S5) and resistance classes (Table S6). The alignment was analyzed using Resistome Analyzer (commit 15a52dd to github.com/cdeanj/resistomeanalyzer) followed by normalization using reads per kilobase million per gene. This workflow is implemented as part of the CAP pipeline (Danko et al. 2021).

### Genome mining

Biological Gene Clusters were identified using the BioSynthetic Spades option (“spades.py –bio”) within the Spades assembler (Bankevich et al. 2012) (v3.14.1). This option produces an assembly of the sample sequences (scaffolds.fasta) as well as an assembly enriched only for the classes of non-ribosomal (NRPS) and polyketide (PKS) gene clusters (gene_clusters.fasta). As we are looking to identify BGCs across all potential classes, we proceeded with the full assembly (scaffold.fasta) for the subsequent steps. This analysis was performed separately for each sample. The standalone version of AntiSMASH5 (v5.2.0) (Blin et al. 2019) was used for identification of BGCs in the assembled scaffolds, as well as their classification corresponding to specific types of SMs, and annotation based on similarity to entries in the database of BGCs with known output compounds MIBiG2 (Kautsar et al. 2020). We used prodigal (Hyatt et al. 2010) for gene finding and concentrated on bacteria as taxa. Full specified settings are “—genefinding-tool prodigal—taxon bacteria—cb-general—asf—smcog-trees—cb-knownclusters—cb-subclusters—pfam2go”. This analysis was also performed separately for each sample.

We combined the AntiSMASH output from all the samples into a list of all BGCs identified across all samples, and used BiG-SCAPE/CORASON (Navarro-Muñoz et al. 2020) to generate gene cluster families according to structural characteristics (using similarity networks) and sequence characteristics (using the Pfam directory). We also used Big-SCAPE to also annotate the families which encode a product similar to the known products in MIBiG2 using the “—mibig” setting.

## Results

### Microbial community characteristics as related to environmental context

#### Microbial community metrics

**Figure 2.**
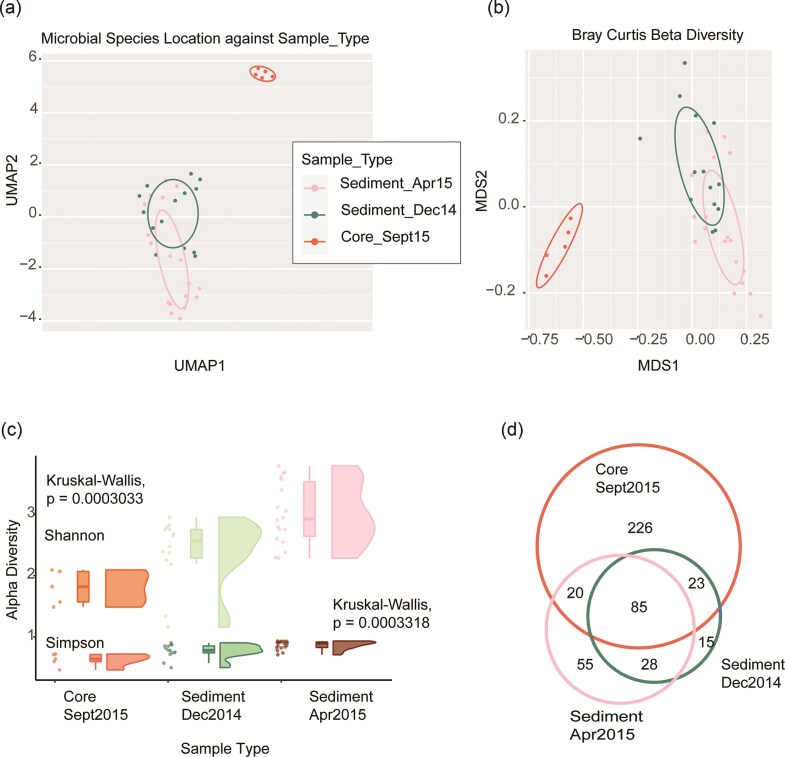
(a) UMAP dimension reduction visualization of taxonomic profiles based on MetaPhlAn3 species abundance profiles. Color legend is shared with Fig. 2b. Axes are arbitrary and without meaningful scale. (b) NMDS visualization of Beta Diversity using the Bray–Curtis dissimilarity metric separating sample sets. Ellipses represent 99.99% confidence intervals. (c) Alpha Diversity according to Shannon and Simpson indices. Each plot includes a scatter plot, a box plot, and a half violin plot. (d) Venn Diagram of species identified across different sample sets.

Species abundance was computed for all the samples. Kendall correlation (Fig. S1) shows groupings of Gowanus samples into two broad sets, the Core Samples and the Sediment Samples. UMAP dimensional reduction (Fig. 2a) reflects a differential distribution of species based on both location and data type, and groups the samples as Core, Sediment_Winter14 and Sediment_Spring_15, with an overlap between the two Sediment samples. In order to investigate sequencing depth as a factor in this grouping (Core samples were sequenced at higher depth that the Sediment samples, see Table S1 for read depths), we downsampled the Core reads to the average Sediment read depth to generate a normalized dataset. This normalized dataset also generates a similar UMAP plot as seen in Fig. S2, and the full dataset was used for all subsequent analyses. Of a total of 455 species identified across all samples (Table S2), we identified including 13 archaeal species, 241 species of bacteria, and 201 viruses/phages. Of these, 85 species are shared across all the 3 sample sets, and the 2 sediment sample sets share 128 species (Fig. 2d).

We next evaluated intra-sample alpha and beta taxonomic diversity of our collections. We found that samples from the Core set were different from Sediment samples with lower alpha-diversity (Fig. 2c). While the Shannon index accounts for species richness by including rare species, the Simpson index gives more weight to evenness and common species. Both metrics are independent of group size, therefore the uneven distribution of sample numbers in Core and Sediment sets does not affect the alpha diversity metric (Roswell et al. 2021). Shannon [Kruskal–Wallis, chi-squared = 16.202, *P*-value = 0.0003033] and Simpson indices [Kruskal–Wallis, chi-squared = 16.022, *P*-value = 0.0003318] attain statistical significance. Beta-diversity was calculated by Bray–Curtis dissimilarity index and scaled using NDMS (Fig. 2b). The NDMS plot of the taxonomic profiles showed slight separation between the two groups of Sediment sample sets; however, these differences were minor. The samples in the Core set are different from the samples in the Sediment set according to this metric, also previously observed in the UMAP plot (Fig. 2a).

#### Species characterization according to environmental and historical context

##### Marine organisms related to geographic environment

Originally in a productive tidal estuary stewarded by the Lenni Lenape people for over 9000 years before colonial contact (Doyle 1996), the present-day environment of the canal is a combination of original sediment, over 150 years of industrial waste, inputs from surrounding runoff and combined sewage overflows (CSOs), and tidal influx. Accordingly, many species found in the Gowanus Canal were previously identified in either marine or freshwater sediment, water or soil. (See Methods for microbial characterization strategies.) Marine sediment species identified include *Modestobacter marinus* [also radiation-tolerant (Normand et al. 2012)], *Labilibaculum filiforme, Draconibacterium orientale* (Du et al. 2014)*, Maribacter arcticus, Sedimentitalea nanhaiensis, Halioglobus lutimaris, Shimia aestuarii*, and *Thiobacillus denitrificans*; and *Planktomarina temperata* from sea water. Fewer freshwater sediment species were identified, including *Methanobacterium lacus* and *Xanthobacter autotrophicus*. Known soil microbes identified include *Micrococcus aloeverae* and *Mycolicibacterium chubuense*.

##### Extremophiles related to contamination

Given their high concentrations of contaminants, Superfund sites are considered to be extreme environments (Tighe et al. 2017). We identified a number of extremophiles in the Canal, including psychrophilic, thermophilic, and halophilic organisms. Psychrophiles (cryophiles) are organisms capable of surviving in low temperatures especially in deep sea beds and sediments. We identified psychrophiles such as *Cryobacterium arcticum* (Bajerski et al. 2011), *Desulfobacter hydrogenophilus, Hyphomonas jannaschiana* (also a barophile), *Labilibaculum filiforme, Ma. arcticus*, and *Thiobacillus denitrificans*. The EPA and the New York Department of Environmental Conservation has classified Gowanus as Class SD type saline environment (GEI Consultants 2009). Consistent with this, we observed halophiles, defined as organisms tolerant to hyper saline environments, including *Desulfobacterium autotrophicum, Mi. aloeverae* (also an alkaliphile), *Se. nanhaiensis*, and *Vibrio diazotrophicus*. We also observed a few thermophiles which are organisms adapted for survival in higher temperatures such as *Lachnospira pectinoschiza* and *Thermoleophilum album*. On a species level, some of the extremotolerant organisms identified are known for their bioremediation properties. *Microbacterium laevaniformans*, which has been identified across multiple samples, has previously been isolated from areas contaminated with heavy metals (Brown et al. 2012).

##### Microbial species related to the human gut

Consistent with the frequent CSO from the 11 sewage outfalls (see report “Gowanus Canal—DEP” by U.S. EPA 2025) that can release untreated waste into the Canal, we have identified species usually associated with the human gut. In the following list of species, those known for their potential for developing resistance to clinically used antimicrobial drugs are starred (*). These include Gram-negative bacteria like *Acinetobacter johnsonii, Adlercreutzia equolifaciens, Gemmiger formicilis*, and *Tyzzerella nexilis* and Gram-positive human gastrointestinal bacteria, including Clostridium leptum, Anaerostipes hadrus, Blautia obeum, Holdemanella biformis, Bulleidia extructa*, Collinsella aerofaciens*, Collinsella intestinalis*, ​​Enorma massiliensis*, Gardnerella vaginalis*, Mycobacterium gordonae*, Lach. pectinoschiza*, and Streptococcus salivarius*. Lactobacillus animalis* also isolated from the Canal, is a commonly consumed probiotic. Archaeal commensal species known to be components of the gut microbiome were identified, including Methanobrevibacter smithii* and *Methanobacterium formicicum*. See Table S3 for annotation of microbial characteristics generated through the Microbe Directory (Sierra et al. 2019).

#### Gowanus canal as a reservoir of antimicrobial resistance

Investigating further the potential for the Gowanus Canal to be a reservoir of AMR, we characterized the known classes and mechanisms using the database MEGARes (Table S4 for list of genes associated with AMR, Table S5 for their mechanisms and Table S6 for their classification). We identified 28 genes within 8 classes of antibiotic resistance, the major ones being Rifamycin (which includes Rifampin) and Aminoglycosides, and others, including Tetracyclines, Elfamycins, Phenicol, Sulfonamides, Multi-Drug Resistance, and MLS as shown in Figure S3a. Of these, the most frequently identified gene (ARO:3 003 465, confiring rifampicin resistance) has been identified in 15 out of the 37 samples. The common drug-class mechanisms through which the above noted genes confer resistance is shown in Figure S3b.

The above analysis attributes genus-level classification when possible to the identified genes, and in this analysis, two of the AMR genes were attributed to Mycobacterium (one Aminoglycoside and one Rifampin, Table S4). While we might assume that these AMR all come from human gut species as those flagged above, some environmental species identified in this dataset are known to harbor resistance genes as well. These include species previously isolated from water and sediment such as the sulfate-reducing *Desulfobacter hydrogenophilus* and *Desulfobacterium autotrophicum*, as well as *Thermoleophilum album, Se. nanhaiensis, My. gilvum*, and *My. fallax*. Other species potentially harboring AMR have been previously isolated from soil (*My. chubuense* and *My. obuense)*, and plants (*Mi. aloeverae*) (Prakash et al. 2014).

### Characterization of bioremediation metabolisms

#### Bioremediation pathways overview

**Figure 3.**
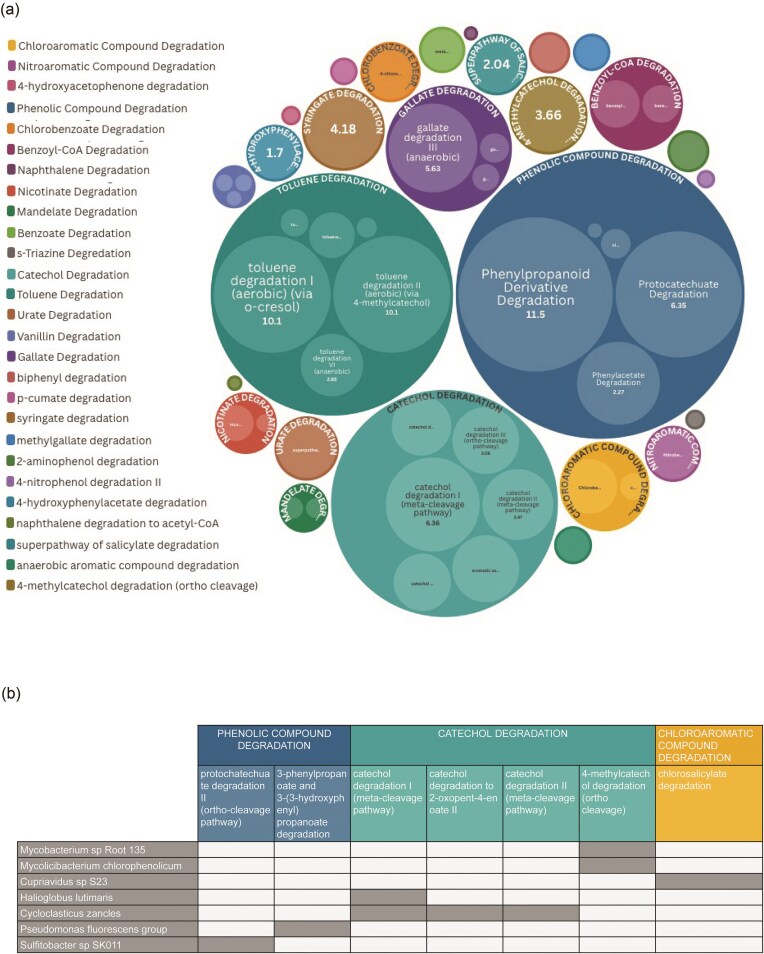
(a) Relative abundance of metabolic pathways identified across all samples related to aromatic compound degradation (within the sub-class of DUA). (b) Correlation plot depicting the subset of pathways within the class of Aromatic Compound Degradation that were attributed to specific species.

To further understand the relationship between the Canal microbiome and its environment, we classified metabolic pathways as defined by the MetaCYC (Caspi et al. 2020) database using the software HumanN3 (Beghini et al. 2021), (Table S7 for pathway list). Among them, we identified 64 pathways related to the Canal’s history of contamination, using the list of biotransformation pathways compiled by EAWAG (Gao et al. 2010) as keywords. Among these, 61 pathways fall within the MetaCyc superclass of Degradation/Utilization/Assimilation (DUA), two pathways belonging to the Biosynthesis superclass and one in Detoxification (Table S8). Within the DUA superclass, Aromatic Degradation is the most represented with 52 pathways, including compounds of historical relevance such as toluene, cresols, catechol and phenolic compounds (Fig. 3a).

#### Bioremediation pathways of historical relevance and potential biotech applications

##### Aromatic compound degradation

Within the Aromatic Degradation class, toluene degradation is the most prevalent subclass (28%), followed by catechol (23%) and phenolic compound degradation (12%) (Fig. 3a). Seven of the bioremediation pathways were attributed to specific microbial species (7 species) (Fig. 3b). In two cases, we observe a one-to-many relationship: a given pathway is attributed to multiple species (e.g. “4-methylcatechol degradation” is associated to *My. chlorophenicum* and *Mycobacterium sp Root135*) and in one case, a many-to-one, i.e. a given species is associated with multiple pathways (e.g. *Cycloclasticus zancles* is associated to “catechol degradation to 2-oxopent-4-enoate II”, “catechol degradation I”, and “catechol degradation II”). In three cases, we observe a one-to-one relationship.

Most of the pathways identified here are related to compounds identified by the EPA Superfund study (see report “Gowanus Canal Site Profile” by U.S. EPA 2025); however, some metabolisms relate to compounds that are not reported in this assessment, such as the PAHs mandelate and gallate.

Besides aromatic compound degradation, the DUA pathways identified also fall in the C1 compound (Lee et al. 2019) utilization and alcohol degradation classes. Relevant to historical contaminants, these include formaldehyde assimilation, also not listed in the EPA site profile report.

##### Heavy metal superstar

In addition to organic contaminants, we assessed the metabolic potential of the Gowanus Canal microbiome for response to inorganic contaminants, specifically heavy metals.

Of the 2 808 569 total genes identified by HumanN3 (available at https://github.com/HenaffLab/2023_Gowanus_Publication), 3153 encode proteins that are associated with heavy metals, with the largest number of genes associated with Iron (1743 genes) followed by Cobalt (446), Copper (441), and Nickel (286). Cadmium, Chromate, Lead, Mercury, Vanadium, Arsenic, and Aluminum all counted <100 genes for a given metal. Mechanisms include resistance (141 genes), oxidation/reduction (299), chelation (88), efflux (43), transport (939), and binding (752) (Table S10).

Of the 3153 total metal-associated genes, 1172 are associated with one or more of 201 unique bacterial species (Table S12). Some of these species are associated with a large number of these genes, covering multiple metals (top species are *Desulfobacterium autotrophicum, Methanobacterium lacus, Desulfococcus oleovorans, Ruminococcus torques, Desulfobacter hydrogenophilus, Methanosarcina_sp_MTP4*, each encoding more than 20 metal related genes).

### BGC discovery

Our approach to BGC discovery differs from the sequence similarity based functional analyses described above, since our method (1) identifies gene cluster *structures* in genomic data, as well as the sequence characteristics, and (2) utilizes assembled scaffolded contigs, rather than using single reads.

SM compounds were divided into six classes depending on their biosynthetic origin: polyketides (PKs), non-ribosomal peptides (NRPs), Ribosomally synthesized and post-translationally modified peptides (RiPPs), terpenoids, saccharides, and indole alkaloids (Keller et al. 2005, Medema et al. 2015). Overall, we identified 2319 assembled BGCs in our 37 total samples, including both sediment and core samples [using AntiSMASH5 (Blin et al. 2019) on assembled contigs, see Methods for more details]. Of the 2319 assembled BGCs, 1600 (69%) were classified into 5 predominant classes [using Big-SCAPE (Navarro-Muñoz et al. 2020), which represents a broad range of structural classes], with the remaining 719 unclassified (31%). The SM classes identified include Polyketides (PKS1 and PKSOthers, 18%), Ribosomally synthesized and post-translationally modified Peptides (RiPPs, 21%), followed by nonribosomal peptide synthetases (NRPS, 15%), Terpenes (14%), and PKS-NRP Hybrids (<1%).

**Figure 4.**
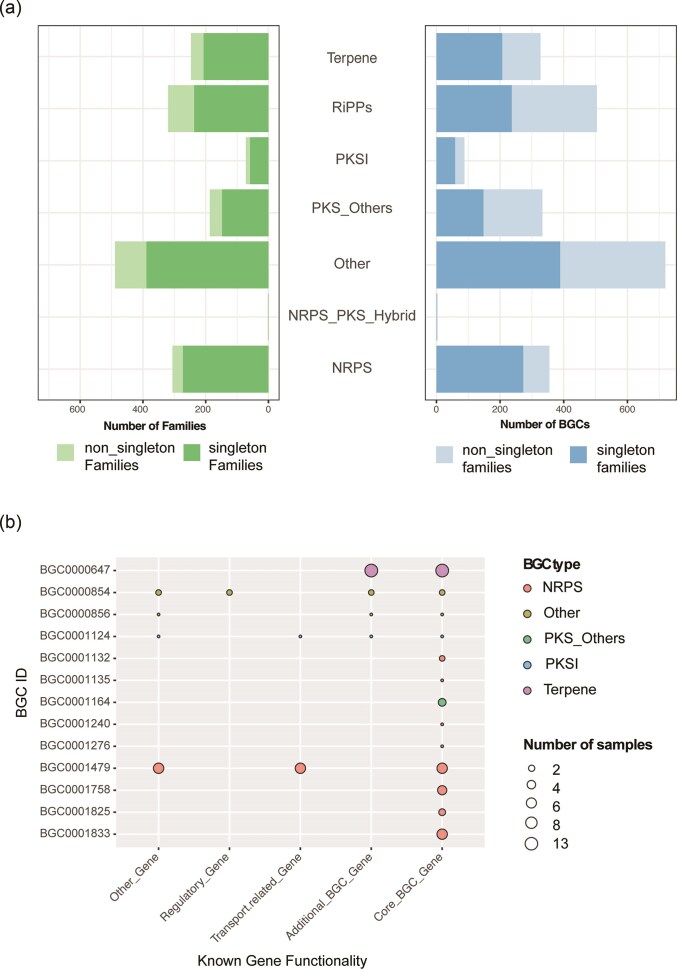
(a) Stacked bar plot representing the number of BGC families by BGC class, differentiating singleton and non-singleton families. (b) Subset of gene cluster families characterized according to the MIBiG reference. Circle size depicts the number of BGCs belonging to the family, the color represents the type of BGC, position along the *X* axis depicts functional genes identified.

BGCs can be grouped into gene cluster families (GCFs, or “families” hereon) that are functionally closely related and encode the production of the same or very similar molecules, which Big-SCAPE characterizes according to structural and sequence characteristics. Of the 2319 BGCs identified in our samples, 44% (1005) exhibited homology with at least one other BGC, grouping into 309 families, while 56% (1314) were singletons (considered as 1314 families of one). These total to 1623 families defining the 2319 total gene clusters identified (Fig. 4a). The largest family counts 28 BGCs, found in the group PKSOthers, followed by 12 in the RiPPs group. The average number of BGCs per family is 1 or 2 in every group.

While many families of BGCs have been computationally defined, few have been associated with specific SM compounds experimentally. The Minimum Information about a BGC (MIBiG 2.0) database (Kautsar et al. 2020) records such BGCs with known outputs, and among the 1623 families in our dataset, 13 of these display high similarity (>80%) with BGCs in MiBIG (calculated as the % of genes in the query cluster that have significant BLAST hits with a gene in the target cluster) (Fig. 4b). The remaining 1610 are not associated with a specific compound, and could potentially encode unknown (novel) compounds.

## Discussion

Here, we present the first in-depth metagenomic study of the Gowanus Canal, a Superfund site embedded in a residential/light manufacturing neighborhood of Brooklyn, NY, USA. Taxonomic classification shows that this contaminated environment hosts a rich and diverse microbial population. The composition of this community includes species previously identified in marine sediment, as well as the human gut, reflecting both the estuarine environment and the presence of CSO outfalls.

Collectively, the microbiome of the canal encodes a wide range of antimicrobial resistance genes, including some related to human pharmaceutical use. While some AMR alleles come from human gut species, some environmental species identified in this dataset are also known to harbor resistance genes. These include species previously isolated from water and sediment such as the sulfate-reducing *Desulfobacter hydrogenophilus* and *Desulfobacterium autotrophicum*, as well as *Thermoleophilum album, Se. nanhaiensis, My. gilvum*, and *My. fallax*. Other species potentially harboring AMR have been previously isolated from soil (*My. chubuense* and *My. obuense)*, and plants (*Mi. aloeverae*). Of note, the material sampled in this study was sediment and this substrate contained both human gut related microbes as well as human related AMRs. This means that to a certain extent, human gut microbes from untreated waste streams are persisting in the sediment. Further research could address the fate of waste stream related microbes in the environment, their interactions with chemical contaminants, and the potential of CSOs to develop environmental AMR reservoirs. Recently literature has accumulated that points to contaminants’ effect on ARG persistence in the environment (Fletcher 2015), including historical industrial contaminants as well as emerging organic contaminants (Alderton et al. 2021). One such mechanism for this impact is that the prevalence of ARGs carried by live bacteria is impacted by the selection pressures their hosts are subject to (Mathur et al. 2018).

Microbes evolve faster under stress (Li et al. 2014), and the Canal microbiome has been challenged with industrial contaminants since before the canal was dredged in 1853. We assessed the potential for this microbiome to be a reservoir of remediation-related functions through functional analysis, identifying gene coding potential and metabolic pathways encoded in this dataset. We identified bioremediation pathways related to degradation of VOCs and complex hydrocarbons, consistent with the historical industries of coal extraction and paint making at the site (Miller 2015). Interestingly, our data suggests that a given microbial species usually uses multiple mechanisms for degradation of a particular aromatic compound, and targets just one group of hydrocarbons. For example, *Pseudomonas fluorescens* encodes degradation of catechol through three pathways, including meta-cleavage and through the intermediate 2-oxoent-4-enoate. We also identified gene interactions related to heavy metals. In the case of metal related functions, a given species may encode molecular functionality related to multiple metals. Top species are *Desulfobacterium autotrophicum, Methanobacterium lacus, Desulfococcus oleovorans, Ruminococcus torques, Desulfobacter hydrogenophilus, Methanosarcina_sp_MTP4*, each encoding more than 20 metal related genes. While heavy metals are essential cofactors in many cellular processes, high heavy metal concentrations are also a stressor and microbes adapt by developing resistance mechanisms, including chelation and efflux (Anastassopoulou and Theophanides 1995, Lemire et al. 2013, Oves and Khan 2016, Panda et al. 2021). As such, these resistance mechanisms can be applied to biotech applications such as remediation through biosorption and bioaccumulation (Chojnacka 2010). Additionally, there is an interesting potential for using microbial mechanisms to recover rare earth metals from contaminated environments, as these metals are both contaminant and valuable resource (Jyothi et al. 2020). These studies encourage further research to gain insight on the biochemical potential of Superfund sites from the perspective of remediation and resource recovery.

The current gold standard for characterization of new SMs is through metabolomic discovery. However, recent efforts to identify useful SMs leverage computational methods to mine metagenomic datasets for BGCs. These efforts are concentrating on soils as environments with potential for pharmacologically relevant molecules (Charlop-Powers et al. 2016, Negri et al. 2022, Waschulin et al. 2022) and on human body environments with potential to relate to disease phenotypes (Donia et al. 2014, Aleti et al. 2019). Here, we show that the Gowanus Canal depicts its own microenvironment encoding novel BGCs. This distribution of BGC families mirrors that reported by Nayfach et al. (2021) for the “bioremediation” category, as compared with other sources including aquatic, wastewater or terrestrial environments. The BGC family distribution observed in the Gowanus, however, differs from that reported in another Superfund site in Vermont, the Ely Copper Mines (Giddings et al. 2020). Indeed, the top two classes of the BGCs in Ely Copper Mines included NRPS and Terpenes. Of note, water and sediment can often have separate BGC profiles such as those described by Nayfach et al., and those from Lake Hillier, a hypersaline environment with distinctive pink color (Sierra et al. 2022). This signifies that irrespective of similarities between multiple extreme environments, the BGC characterization of each environment is unique.

The results detailed in this manuscript point to the microbiome of the Gowanus Canal, an urban EPA Superfund site, as a reservoir for microbial mechanisms potentially important for biotechnological applications. Indeed, these microbes harbor bioremediation functions, degrading complex hydrocarbons as well as fixing heavy metals while also surviving in extreme conditions as a complex community. These functions can be seen as a resource, and further studied for potential biotechnological and engineering applications. One can imagine at least two avenues for augmenting the bioremediation potential of this resident microbiome. On one hand, one can isolate and characterize specific strains with functions of interest and culture them in bioreactors (Navina et al. 2024), perhaps engineering them to overexpress these genes for accelerated degradation (Dangi et al. 2019). This can be implemented in treating isolated waste, but poses challenges when scaling up and/or releasing genetically engineered strains into the environment (Navina et al. 2024). On the other hand, one can consider altering the *environment* in which these consortia have evolved, to help overcome limiting steps or apply principles of directed evolution. This has been successfully implemented in the context of microbial treatment of wastewater (Saravanan et al. 2023a), and biochar has been shown to effectively augment bioremediation performance due to its properties in altering microbial habitats on a micro scale (Saravanan et al. 2023b). Moving forward with either of these options requires considering the ethics of interventions on non-human organisms, because even though we can’t relate to microbes in the way we can to beasts of burden, “harnessing” them still implies making resources through labor (Krzywoszynska 2020).

This work shows the co-occurence of ARGs and bioremediation metabolisms, and exciting future perspectives emerge from this research. In particular, an open question posed by this work and to be addressed in future studies is: what is the impact of industrial contaminants on the persistence of antimicrobial resistance genes in environmental reservoirs? These results outline the potential of this Superfund site and other contaminated sites across the world to be case studies to uncover the mechanics of interactions between antimicrobial resistance, environmental contamination, and microbial community dynamics. We anticipate to follow up with this work with long-read sequencing approaches, contaminant quantifications, and co-culture experiments that would be able to reveal interactions within the consortia and their impact on metabolisms and ARG persistence.

## Conclusion

Once an ecologically diverse estuary and home to the Lenni-Lenape people, the Gowanus Creek was dredged in 1853 as part of ongoing colonial and industrial disruption to the land to become the Gowanus Canal. Its environment today is emblematic of the many post-industrial Superfund sites across the country. Like Gowanus, many of these locations were important sites for production and manufacturing industries that have now moved elsewhere leaving a material, economic, and social legacy of toxicity not only in the canal itself but also the surrounding areas As a result of the EPA’s remediation proposals (U.S. EPA 2012), the communities surrounding the Gowanus Canal (notably, those historically disproportionately affected by the contaminated environment) are now vulnerable to residential displacement, and deepening economic inequality (Krisel 2015) in addition to ongoing possible toxic exposure.

The Gowanus Canal is considered a forsaken environment from our human perspective: the Superfund cleanup plan is to dredge the canal and cap it with an impermeable layer. Yet, this study shows that this environment is thriving from a microbial perspective. These microbes have evolved to survive harsh conditions, and have shown capability of not only remediate contaminants in the environment, but also produce novel SMs.

These data show there is still much we can learn from these lifeforms who have evolved to inhabit industrial ruins. At a time where human disturbance has become a geological force, these results further suggest that we should redefine our relationship to the lifeforms we live amongst, with a responsibility for stewardship and reciprocity. These microscopic neighbors not only serve as a genetic catalog of bioremediation functions, but open our imagination to new modes of what theorist Anna Tsing calls “collaborative survival”—of ways to live together on a damaged planet (Tsing 2015).

## Supplementary Material

lxaf076_Supplemental_Files

## Data Availability

Sequence data is available under NCBI SRA Project PRJNA940122 (https://www.ncbi.nlm.nih.gov/bioproject/PRJNA940122). All data generated in the analyses can be found at https://github.com/HenaffLab/2023_Gowanus_Publication
