# Supplementary figures and images for: Metagenomic interrogation of urban Superfund site reveals antimicrobial resistance reservoir and bioremediation potential
Source: J Appl Microbiol. 2025 Apr 16;136(4):lxaf076. doi: 10.1093/jambio/lxaf076 (PMC11999716; doi:10.1093/jambio/lxaf076)

Figure S1

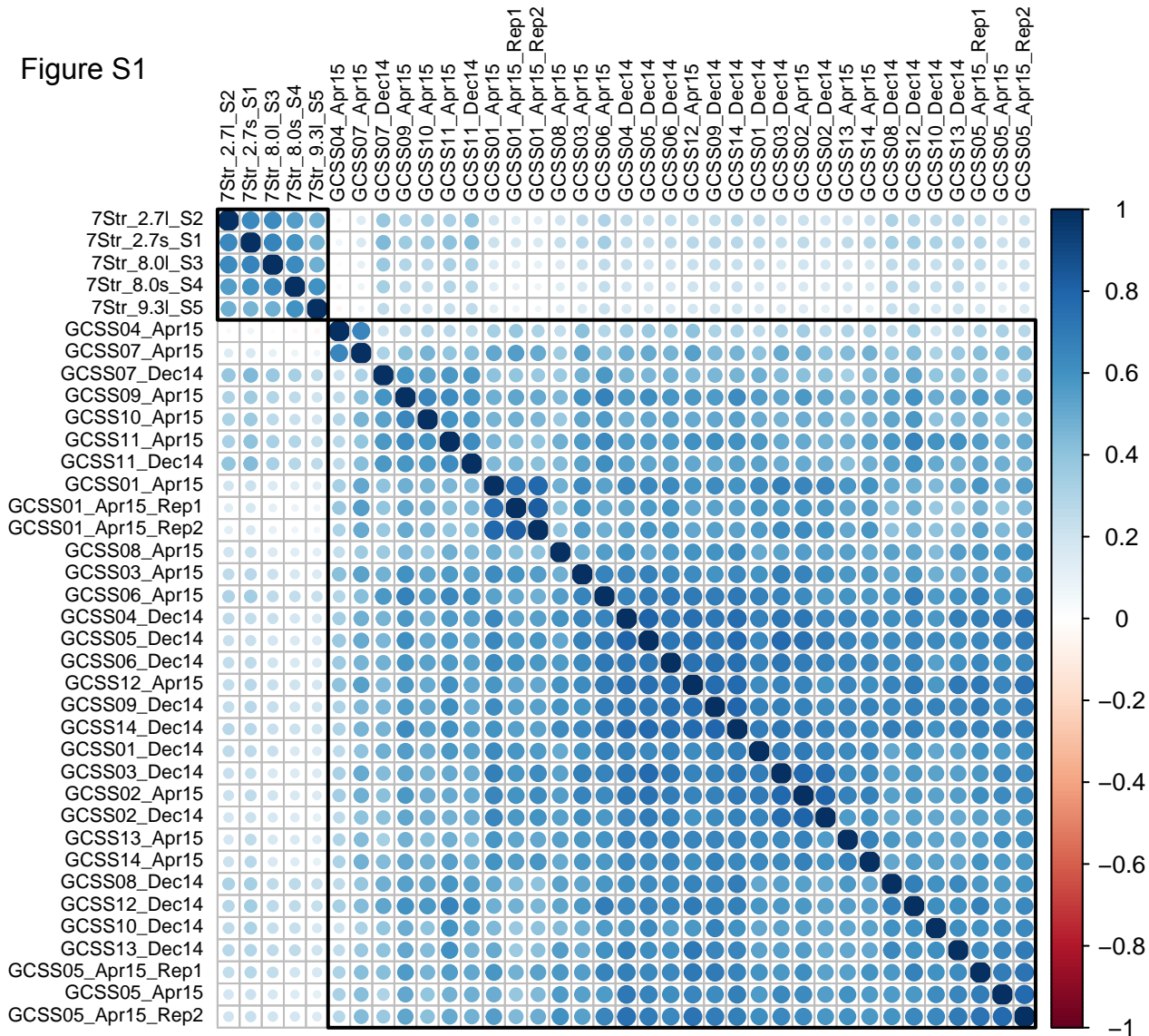

Microbial Species Location against Downsampled Sample\_Type

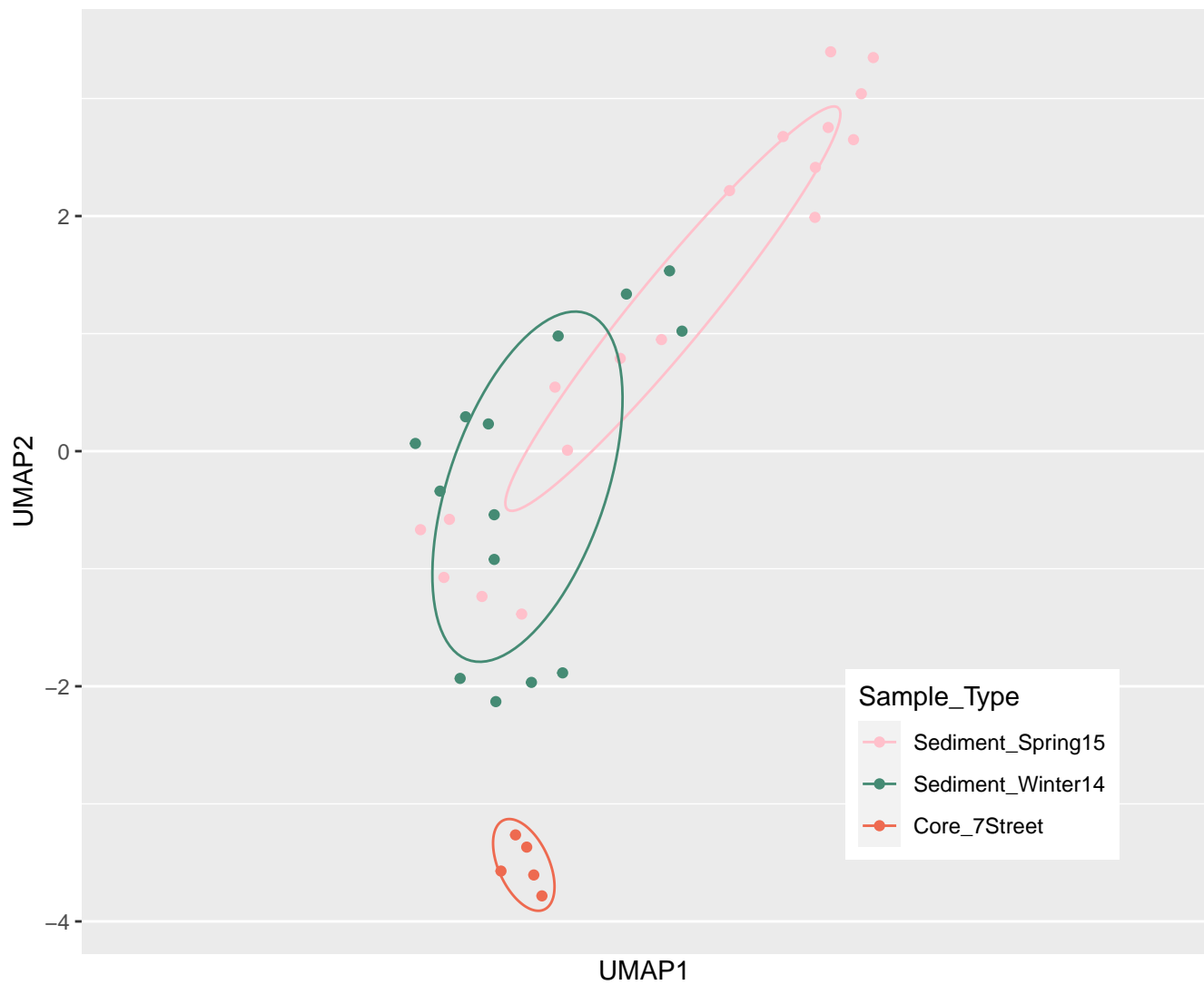

A.

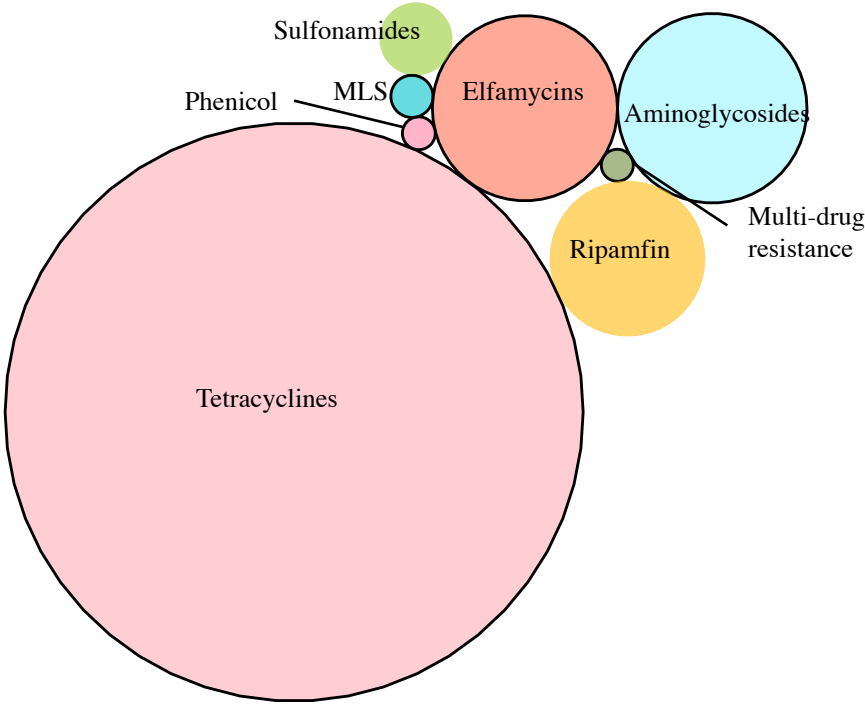

B.

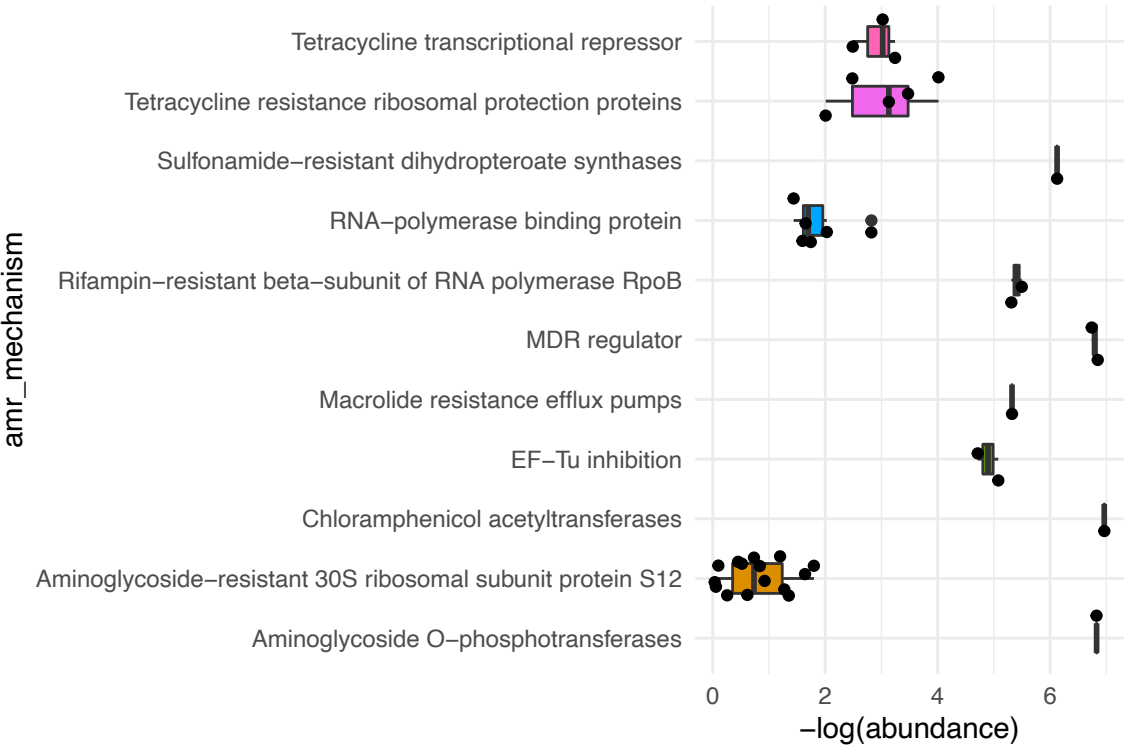

Supplement: lxaf076_Supplemental_Files [file lxaf076_supplemental_files.zip › SuppFig1 - correlation matrix.pdf]

Microbial Species Location against Downsampled Sample\_Type

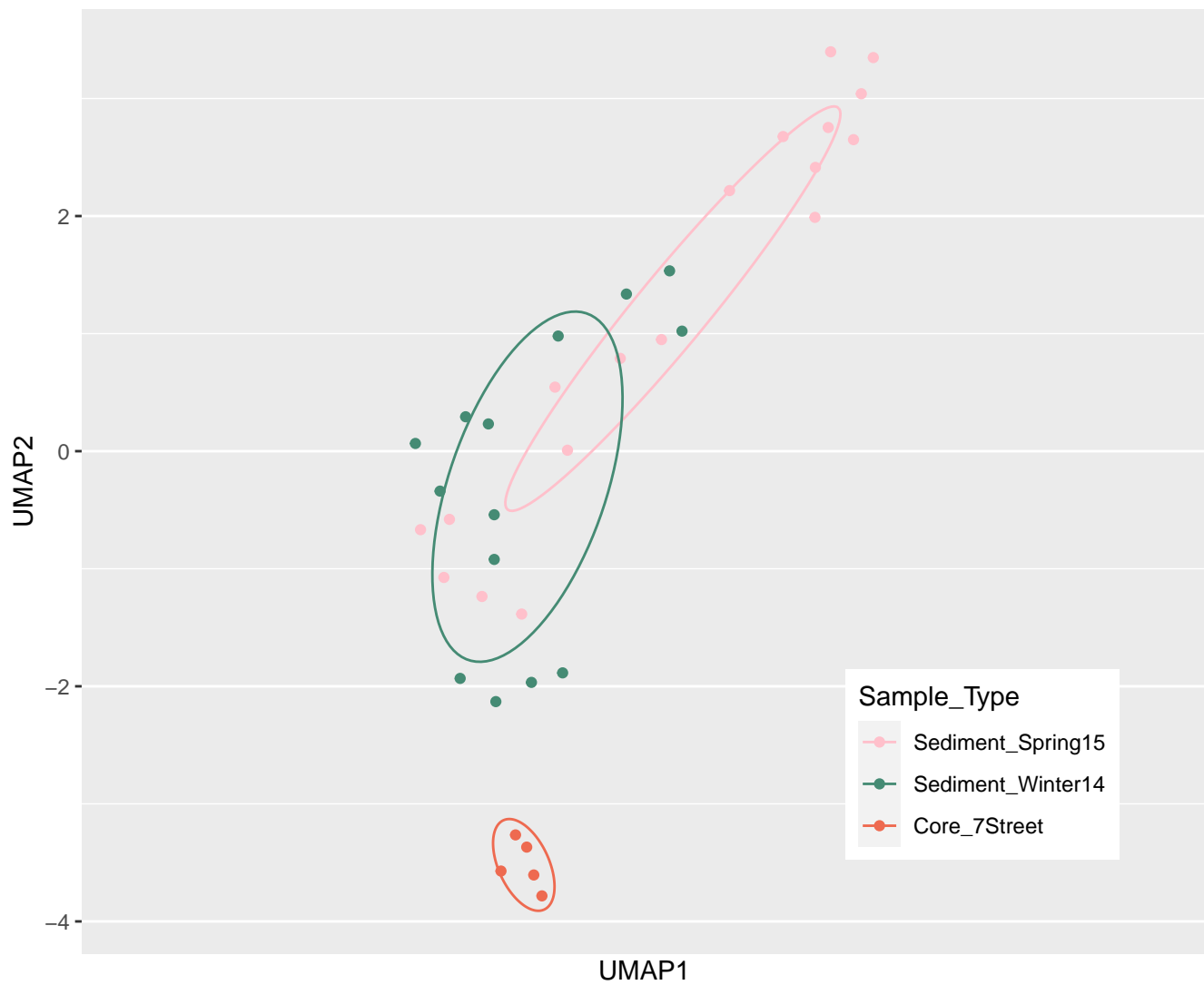

Supplement: lxaf076_Supplemental_Files [file lxaf076_supplemental_files.zip › SuppFig2 - UMAP.pdf]

A.

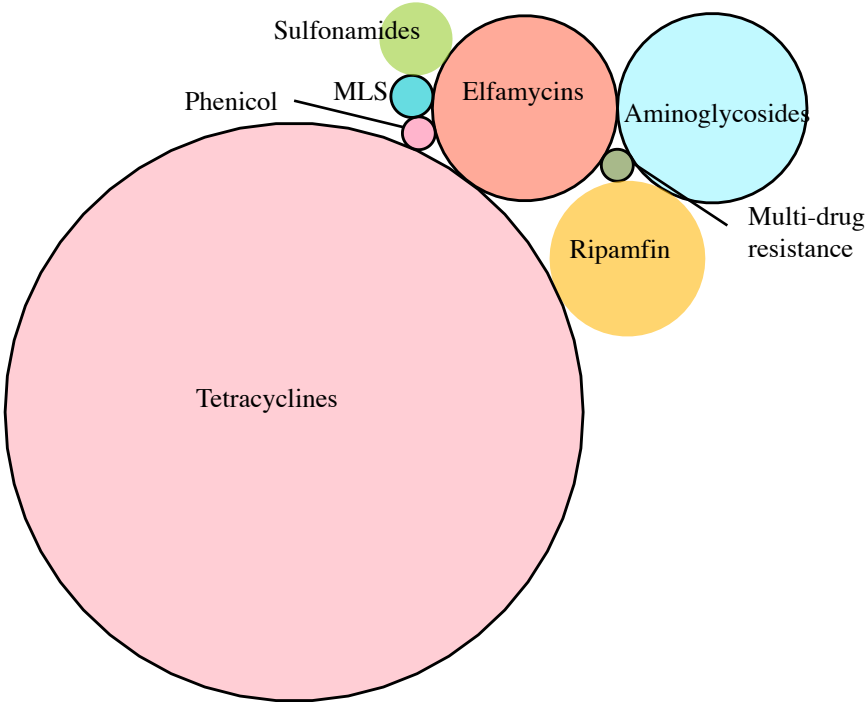

B.

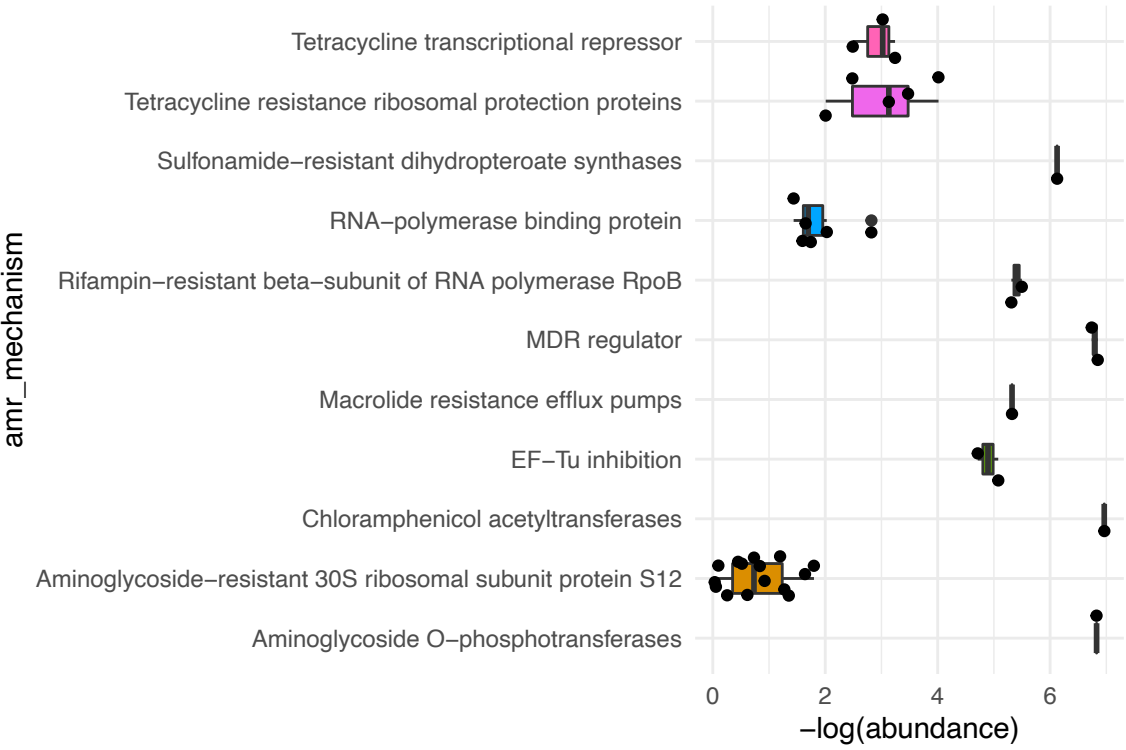

Supplement: lxaf076_Supplemental_Files [file lxaf076_supplemental_files.zip › SuppFig3 - AMR.pdf]
